# Structural characterization of the buccal mass of *Ariolimax californicus* (Gastropoda; Stylommatophora)

**DOI:** 10.1371/journal.pone.0212249

**Published:** 2019-08-07

**Authors:** Devis Montroni, Xiaolin Zhang, Janet Leonard, Murat Kaya, Chris Amemiya, Giuseppe Falini, Marco Rolandi

**Affiliations:** 1 Department of Electrical and Computer Engineering, University of California, Santa Cruz, Santa Cruz, California, United States of America; 2 Departiment of Chemistry “Giacomo Ciamician”, Alma Mater Studiorum Università di Bologna, Bologna, Italy; 3 Joseph M. Long Marine Laboratory, Institute of Marine Science, University of California, Santa Cruz, Santa Cruz, California, United States of America; 4 Department of Biotechnology and Molecular Biology, Faculty of Science and Letters, Aksaray University, Aksaray, Turkey; 5 School of Natural Science, University of California, Merced, Merced, California, United States of America; University of Montreal, CANADA

## Abstract

Biological materials such as chiton tooth, squid beak, and byssal threads of bivalves have inspired the development of new technologies. To this end, we have characterized the acellular components in the buccal mass of the terrestrial slug *Ariolimax californicus* (banana slug). These components are the radula, the jaw, and the odontophore. In the radula, calcium-rich denticles are tightly interlocked one to the other on top of a nanofibrous chitin membrane. The jaw has a nanostructured morphology made of chitin to achieve compression resistance and is directly linked to the foregut cuticle, which has a protective nanofibrous structure. Finally, in the odontophore, we observed a structurally elastic microstructure that interfaces soft tissues with a highly stressed radula membrane. Based on those observations, we discuss the interaction between these components and highlight how the materials in these task-specific components have evolved. This structure-properties-function study of the *A*. *californicus*’ buccal mass may aid in the design and fabrication of novel bioinspired materials.

## Introduction

Biological materials combine exceptional application-specific properties with a combination of structure and composition.[1][2] Examples include the toughness of crustacean shells,[3] the fracture resistance of bones,[4] the damage tolerance of the dactyl club of the mantis shrimp,[1][5] the self-healing properties of byssal threads,[6][7] the stiffness gradient of the Humboldt squid beak,[8] and the structural coloring of butterflies[9] and beetles.[10] These properties have inspired biomimetic approaches to the synthesis of new materials.[11][12][13]

Feeding is essential to life. Many biomaterials with unique properties are related to food processing including apatite from our own teeth[14] and the structure of the chiton tooth, which is the hardest known biological material.[15] Mollusks, except bivalves, start processing their food with the buccal mass, an apparatus very similar in function to the human mouth.[16][17] The buccal mass contains three acellular components: the radula, the odontophore, and the jaw. The radula is a flexible chitinous membrane with tooth-like structures, called denticles, arranged in transverse and longitudinal rows.[18] Chitin is a block co-polymer of β-(1–4)-linked N-acetyl-glucosamine and few N-glucosamine (deacetylated) units and it is the second most abundant biopolymer after cellulose.[19][20] The odontophore is a cartilaginous structure that supports the radula.[21][22] The radula and the odontophore have a complex tissue organization that allows motion[23] and stems from their biogenesis.[24][25] The third component, the jaw, is a chitin- reinforced part of the foregut cuticle, which is located opposite the radula.[26] When a slug feeds on leaves, a leaf is squeezed between the jaw and the radula, which cuts the leaf into pieces.[27]

In many different mollusk species, the radula is the most studied component of the buccal mass.[28][29][30][31][32][33][34][35][36] The radula’s composition and morphology, which is used in taxonomy,[29][30][31][37] varies widely between species depending on feeding habits. For example, the radula of a limpet that scrapes hard surfaces to collect algae has 2–6 large hard cusps biomineralized with iron.[32] In chitons, with similar eating habits, the posterior region of the tooth is instead made of a “softer” material such as apatite.[34] In the radula of the mainly omnivorous slugs of the genus *Ariolimax*, dozens of denticles, that do not contain iron, are present as an adaptation to a softer food.[30][31] In the genera *Haliotis*,[35][36] *Lacuna*,[38] or *Littoraria*[39] radular morphology responds to changes in theirt diet. Similarly, the jaw is smaller in purely carnivorous mollusks than in herbivorous species.[27] Although the jaw has an important role during the feeding process, its structure has been investigated at the micro-scale only externally for taxonomic observations.[26][30][37] The odontophore has been described as a “cartilaginous structure”,[22][24][27] and researches have focused on investigation on its external tissues, which are involved in both the feeding motion and biogenesis of the radula.

**Fig 1:**
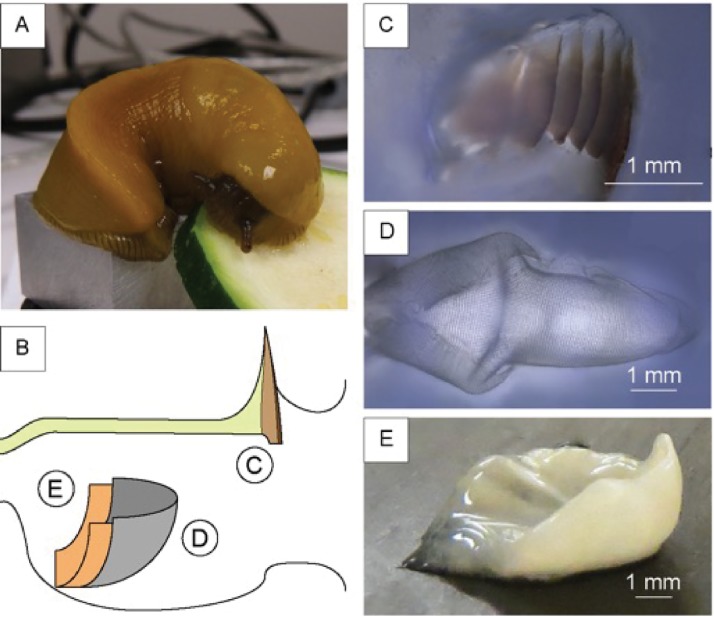
Representation of the components of A. californicus buccal mass. (A) A picture of a banana slug and (B) a schematic representation of the buccal mass; on the left the esophagus opening, on the right the mouth opening. In the scheme we reported the three structures (C) the jaw, shown in the scheme as the brown region while the cuticle is shown in yellow; (D) the radula; (E) the odontophore, dissected out of the surrounding tissues.

The genus *Ariolimax* consists of several different species of terrestrial slugs endemic to western North America, commonly known as banana slugs.[40] These gastropods are omnivores and their diet includes leaves, moss, fungi, dead plant material, and animal droppings.[41][42][43] Here, we have characterized the structure and material composition of the acellular components of the *Ariolimax californicus* buccal mass (Fig 1). There is relatively little information on these components, especially in the buccal mass of terrestrial slugs, or slugs that eat relatively soft food. A better understanding of the structure and composition of the buccal mass may provide insights in how the jaw, radula, and odontophore have evolved in order to adapt to the slug diet.

## Materials and methods

### Sample collection

The material used in this study was derived from a laboratory population of slugs collected at Purisima Redwoods, Half Moon Bay, CA. The slugs were maintained in the laboratory using a protocol previously described.[40] After natural death, we froze the animals for storage and future handling and kept them in a commercial freezer. We collected the three components of interest by dissecting frozen animals after slowly rehydrating them in phosphate-buffered saline (PBS) at 4 °C overnight. Then we cleaned the samples from residual tissues by shaking them in a TWEEN60 solution 5x10^-2^ M for 5 hours[44] and rinsing them with abundant water. We stored the clean samples in PBS at 4 °C.

### Isolation of denticles

We collected the radula’s denticles by putting a radula sample in 500 μL of 8 M urea and shaking it for 24 hours to remove and/or denature the structural proteins in the membrane. Then we treated the radula in 2 mL of hexafluoroisopropanol, a chitin solvent used to soften the radula membrane, and shook it vigorously for 24 hours. We observed a suspension of denticles in solution after the treatment.

### Optic, and fluorescence microscopy images

We treated all the sample sections and the samples reported as “fixed” as follows: the sample was soaked in 2 mL of glutaraldehyde 2.5 vol.% in PBS at 4 °C for 16 hours, washed with PBS, and eventually sectioned using a Leica CM3050S cryo-microtone in 50 μm sections embedding it in O.C.T. Compound by Tissue-Tek, washed with water, and eventually stained before the analysis.

We acquired optical images using a Keyence VHX-5000 microscope and fluorescence images using a Keyence BZ-X710 with a green fluorescence protein filter on CW stained samples.

### Scanning electron microscopy (SEM) images and coupled analysis

We fixed all the samples for SEM using an alcohol gradient (from 10% to 90% every 10%, then 95%, 100% ethanol and 2 x 100% methanol) and critical point drying with methanol/carbon dioxide exchange to preserve morphology. We pre-treated the sample sections and the samples reported as “fixed” as previously reported in the optical microscopy section. We mounted all the samples on a SEM stub with carbon tape, and coated them with 20 nm of gold. We acquired SEM images using FEI Quanta 3D. In the Energy Dispersive X-ray Spectroscopy (EDS) we used an Octane Silicon Drift Detector coupled to a FEI Helios 600i Dual Beam FIB/SEM. We acquired the imaging at 1kV while for EDS we used an accelerator voltage of 20 keV.

### Staining

We performed the Arnow’s assay[8][45] following the following soaking sequence: 1) 100 μL of water; 2) 100 μL of HCl 0.5 M; 3) 100 μL of a solution obtained mixing 10 g of NaNO_2_ and 10 g of Na_2_MoO_4_ in 100 mL of water; and finally in 4) 100 μL of NaOH 1M. We performed a control experiment without adding the sample. After 5 minutes an eventual color change from uncolored to red should occur in the presence of free catechols. We highlighted the presence of chitin using Calcofluor White (CW), a polysaccharide specific stain. We set a drop of stain and then a drop of KOH 10% on the sample, waited 1 minute, and washed away the excess stain with water.

### Spectroscopic analysis

We recorded the Fourier Transform Infrared (FTIR) spectra using a Bruker Vertex 70 FT-IR spectrometer equipped with a diamond crystal ATR accessory. The spectra were collected between 4000–400 cm^-1^ with a 4 cm^-1^ resolution and analyzed using OPUS. The optical scattering properties of chitin were studied with both optical reflection and a transmission-based dark-field microscope (Nikon). The optical spectra were taken using a highly sensitive spectrometer (Ocean Optics HR4000). The specimen was first placed on a glass microscope slide that was later mounted on the stage holder of the microscope. A 20x objective was used to collect the transmitted and reflected scattering signals. The measured scattering light spectra were normalized to the excitation light spectrum.

## Results

We dissected several specimens of *A*. *californicus* (Fig 1A) to analyze the anatomy of the buccal mass (Fig 1B).

**Fig 2:**
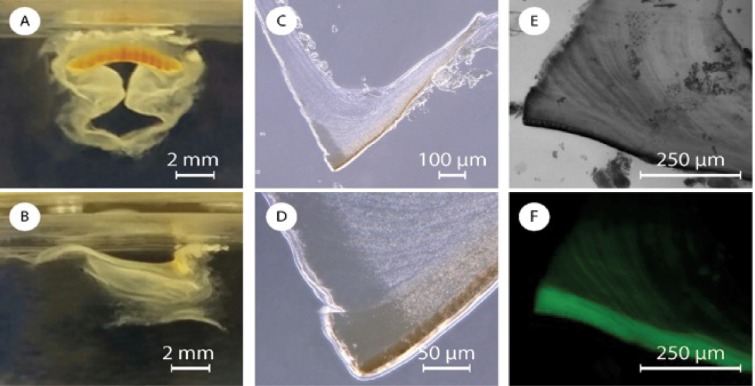
Picture of a jaw in water from the (A) frontal and (B) lateral point of view. (C and D) Optic images of a section of jaw at two different magnifications. Fluorescence microscopy images in (E) bright field and (F) dark field of a section of jaw stained with CW.

We identified in the buccal mass 1) the jaw, 2) the radula, and 3) the odontophore (Fig 1B). The jaw is an anterior stiff brown line of tooth-like structures located behind the upper lip and appeared directly connected with a tunnel-like structure, which covers most of the internal walls of the buccal mass and the esophagus (Fig 2A and 2B) similarly to the peritrophic membrane of insects [46], we will refer to this as the foregut cuticle. The jaw appeared to gradually convert into the foregut cuticle. In *A*. *californicus*, the radula appeared as a rectangular transparent membrane with a triangular end on the short edge below the odontophore, similarly to other mollusks, like limpets[32] or chitons.[34] This transparent membrane carries many denticles on the outer face. In the dissection, we observed the radula lying on top of the odontophore. The odontophore is shaped as a distorted semi-hemisphere and is mostly covered by the radula. While the radula and the jaw are relatively easily separated from the surrounding tissue, the odontophore was strongly connected to the tissue on the edges not covered by the radula (see Fig 1B). We qualitatively observed the jaw, radula, and odontophore working collectively in a living slug observing the slug while eating. During the feeding process, the radula and the odontophore form a structure that scrapes the food by sliding out of the buccal mass to grab a bite. The slug than pinches the food between the jaw’s anterior brown region and the radula allowing the radula to bite off smaller pieces of food.

As part of a more in depth characterization of the jaw (Fig 1C), from analysis of the FTIR spectra, we identified, chitin (Figure A in S1 File) as a major component of the jaw and the foregut cuticle. The presence of chitin was also confirmed by staining with Calcofluor White (CW) (Fig 2E and 2F and Figure B in S1 File). In the FTIR spectra all the absorption bands observed were related to chitin’s vibrational modes. These data did not allow us to discriminate between α- or γ–chitin polymorphs[47][48] (the latter commonly observed in peritrophic membranes[46]). Chitin is white or colorless as a pure material, the jaw, however, is brown (Fig 2A). This coloration might arise from pigmentation, sclerotization, mineralization or chitin-binding proteins. We performed an EDS-SEM (Table A in S1 File) on the jaw and did not find any substantial quantities of minerals, such as iron oxide which may result in brown coloring. To identify the presence of sclerotization, which is usually due to the polymerization of catechols, we used Arnow’s assay, a colorimetric assay, which gave negative results. Overnight HCl 1 M treatment or 2 hours in NaOH 2 M at 80 °C resulted in no color change suggesting that structural proteins, usually hydrolyzed in those harsh conditions, or acid soluble materials are not be responsible for the color.

**Fig 3:**
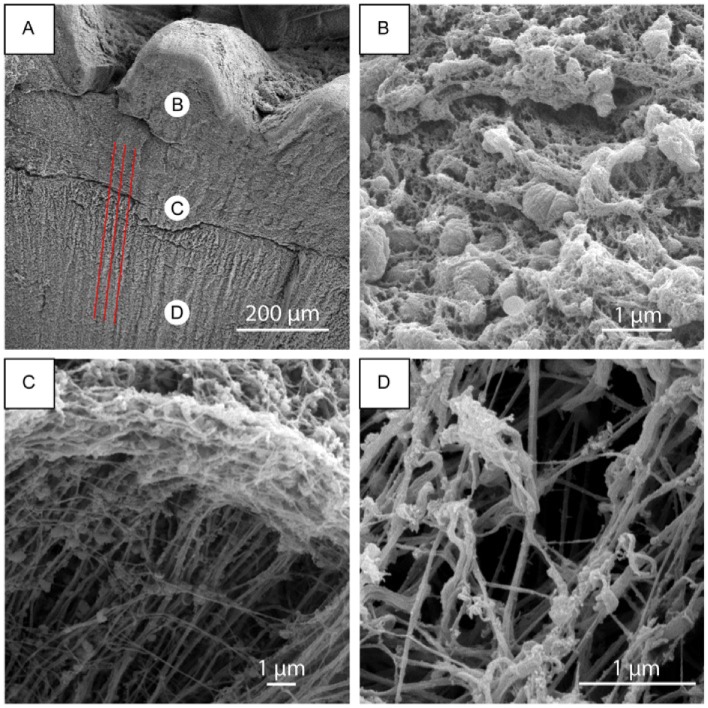
SEM analysis of a jaw. (A) SEM images of a jaw treated in NaOH 1M; red lines highlight the parallel grooves observed in the foregut cuticle. (B) The nano-particles at a higher magnification, (C) a crack of the structure where the perpendicular nanofibers and few particles can be observed, and (D) the nanofibrous carpet.

We thus investigated whether the microstructure may result in structural coloring. The brown structure included a darker layer of 13.8 ± 0.6 μm, in the anterior region, visible in Fig 2C and 2D. This layer becomes lighter inside the tooth-like structure of the jaw and then gradually fades into the foregut cuticle. From SEM images, the jaw is composed of irregular ovoid micro/nano-particles with a length along the longitudinal axis of 800 ± 300 nm (Fig 3A–3D). These particles are densely packed and connected by a network of nanofibrils. In the darker anterior layer the particles appear denser, larger, interconnected, and with few or no nanofibrils (Figure C in S1 File). This external layer is more homogeneous and we were not able to measure effectively the dimensions of the particles. To provide further insights on the potential for structural coloring, we collected both back- and forward-scattering spectra in dark field and the results showed an asymmetrical peak between 400 and 850 nm with a maximum between 550 and 600 nm (Figure D in S1 File) indicating that the structural coloring is likely the cause of the brown appearance.

We then proceeded to investigate the presence of chitin with calcofluoro white (CW). CW staining of chitin in a jaw section indicates the presence of a micrometric fibrous structure (Fig 2E and 2F). In the jaw, these fibers are parallel to the jaw anterior layer (the dense fluorescent area in Fig 2F) and bend closer to the esophagus. The fibers in the jaw continue in the foregut cuticle, where are parallel to the internal wall of the buccal mass. The foregut cuticle is white and does not contain any nanoparticles confirming our conjecture that the nanoparticels provide the jaw with structural coloring. The foregut cuticle instead is made of randomly organized nanofibrous carpet that is a few microns thick (Fig 3B–3D). In Fig 3C, a sample crack allows to see an outer layer of random organized nano-fibers that come from a thicker internal layer of perpendicular nanofibrils.

**Fig 4:**
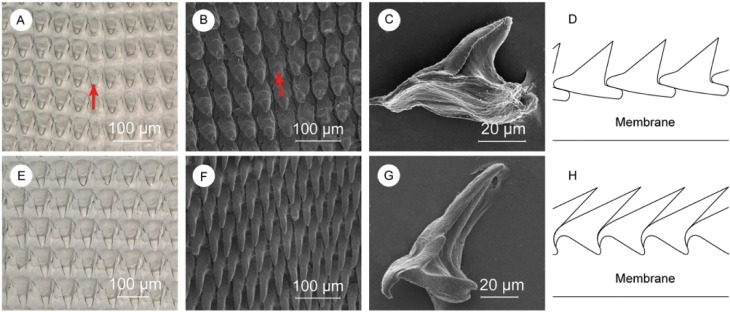
Description of central and lateral denticles. (A and E) Optical microscope images of central, and lateral denticles. SEM images of (B) central denticles, (F) lateral denticles, (C) a single central denticle still showing some fibrils on the base, and (G) a clean single lateral denticle. Schematic representation of the (D) central, and (H) lateral denticles’ packing. Red arrows indicate the rachidian tooth where visible.

**Fig 5:**
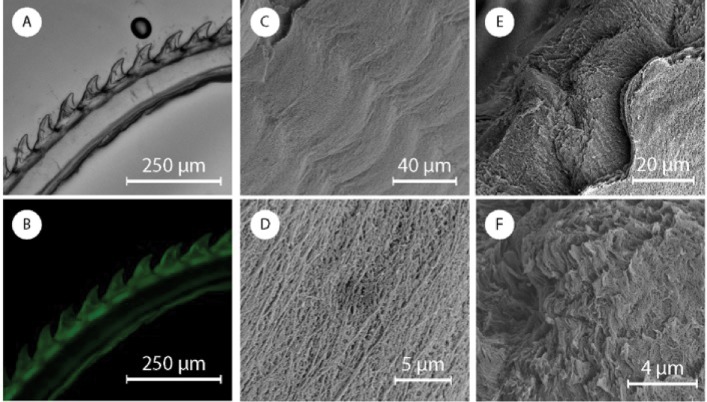
(A) Optical and (B) fluorescence microscope images of a radula longitudinal section. SEM images of (C and D) the radula membrane and of (E and F) a radula membrane section.

We then analyzed the structure and composition of the radula that contains hundreds of denticles packed in rows of approximately 150 (Fig 1D). Perpendicular to these rows are radular ribbons, which are bilaterally symmetrical structures where the central tooth, called a rachidian tooth, is flanked by an equal number of teeth on each side. Denticles have different morphology depending on their position in the radula. Proceeding laterally from the rachidian tooth, denticles increase in aspect ratio (from 1.7 ± 0.3 to 2.9 ± 0.8), length (from 75 ± 9 μm to 93 ± 8 μm), and inclination relative to the membrane (from 37 ± 3 ° to 22 ± 1 °). In 1874, Binney[30] classified these denticles depending on their morphology into two groups: a) central denticles, such as the rachidian tooth as a specific example, with lower aspect ratio and length, and higher inclination(Fig 4A–4C); and b) lateral denticles(Fig 4E–4G). Central denticles have lower aspect ratio and length, while lateral denticles have higher aspect ratio and length. The transition between the two types of denticles occurs gradually across ~10 denticles (Figure G in S1 File). The radula is mainly composed of β-chitin[47][48] (FTIR, Figure F in S1 File) with a minor calcified mineralized phase in the denticles with a calcium signal of about 10 wt.% (EDS, Table B in S1 File). We were not able to detect this mineral phase with FTIR, X-ray diffraction, or micro-Raman spectroscopy. In order to define the chitin micro-structure, we stained a longitudinal section of the radula with CW (Fig 5A and 5B, and Figure G in S1 File). The signal from CW indicated no chitin microstructure in the radula, and a more intense chitin signal was observed in the denticles compared to the membrane. The membrane is composed of an intricate network of nanofibrils with diameters ranging from 300 ± 100 nm (Fig 5C and 5D) arranged in layers (Fig 5E and 5F). These nanofibrils, likely made of chitin, run longitudinally to the radula. An additional fibrous matrix between the larger fibers was dissolved after treatment in NaOH 1 M at room temperature suggesting that this matrix may be made of proteins (Figure H in S1 File). After a urea/hexafluoroisopropanol treatment (see Materials and methods) to weaken the membrane, we collected individual denticles and imaged them with SEM. The central denticles, Fig 4C, did not show any specific anchoring feature. We measured the anterior to posterior distance of the base of these isolated central denticles to be 76 ± 4 μm. When assembled in the radula, the base is 55 ± 4 μm, indicating that there is a certain degree of overlap as sketched in Fig 4D. Lateral denticles (Fig 4G) have a different morphology with a hook on the anterior side of the denticle and a tiny posterior gap. Observation of some radula sections showed how this hook perfectly fit the posterior side of the previous denticle (as sketched in Fig 4H). In a similar way the hook probably fits in the posterior gap.

**Fig 6:**
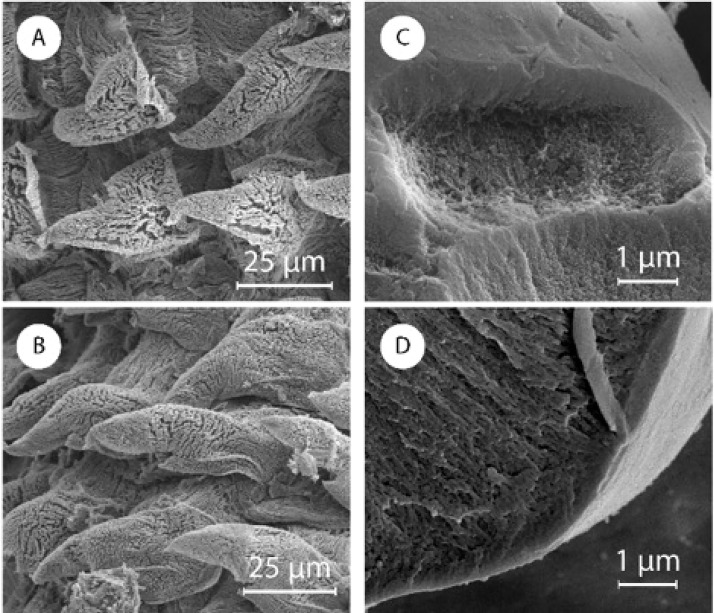
SEM images of immature (A) central and (B) lateral denticles. (C) SEM image of a broken mature etched denticle and of (D) a broken mature denticle.

We found denticles with a completely different morphology on the side of the radula above the odontophore (Fig 6A and 6B). Those denticles were mainly fibrous, with fibers perpendicular to the surface of the denticle, while those in the base were mainly parallel to the membrane. Starting from the most proximal row, the denticles appeared to become progressively more structured until they reached complete maturation. As reported for other species,[24][32] the rows of radular teeth are secreted at the proximal margin of the radula, becoming harder as they mature. The teeth then erode and shed at the distal portion of the radula with the radular ribbon advancing anteriorly to replace rows as they become worn.

CW staining indicated a high concentration of chitin in the denticles, while EDS showed the presence of calcium, but mineralized phases were not detected with standard techniques. We treated a radula in acetic acid pH 3 for 24 hours (Fig 6C and 6D). After the treatment we observed no significant difference on the surface of the denticles. However, some broken denticles showed an external denser layer of 680 ± 90 nm, while the inside appeared as a tightly cross-linked network. An untreated control broken radular denticle showed the same external features with an internal phase that looked mineralized. Finally, as we reported in Figure I in S1 File, a membrane a few hundreds of microns thick, without denticles is present all along the edge of the radula, except where immature denticles were observed.

**Fig 7:**
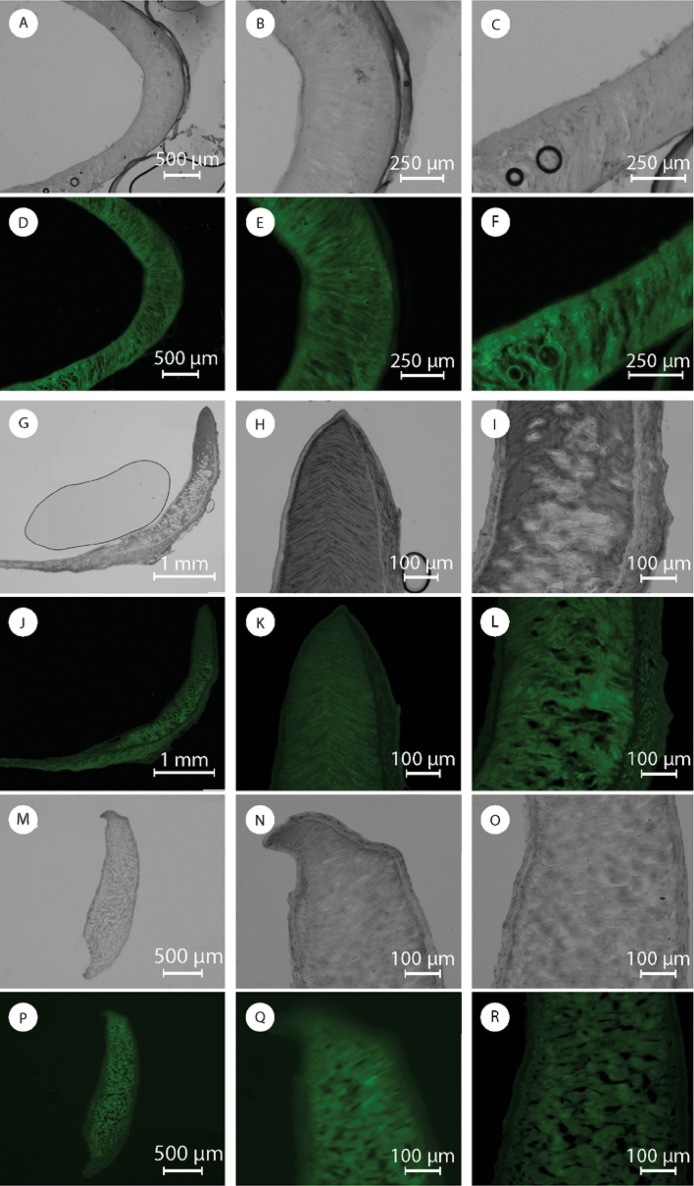
Optical and fluorescence images sections of the odontophore: (A and D) transverse section, (B and E) anterior region in the transverse section, (C and F) posterior region in the transverse section; (G and J) sagittal section, (H and K) dorsal sagittal region and (I and L) ventral sagittal region; (M and P) coronal section, (N and Q) dorsal coronal region and (O and R) ventral coronal region.

**Fig 8:**
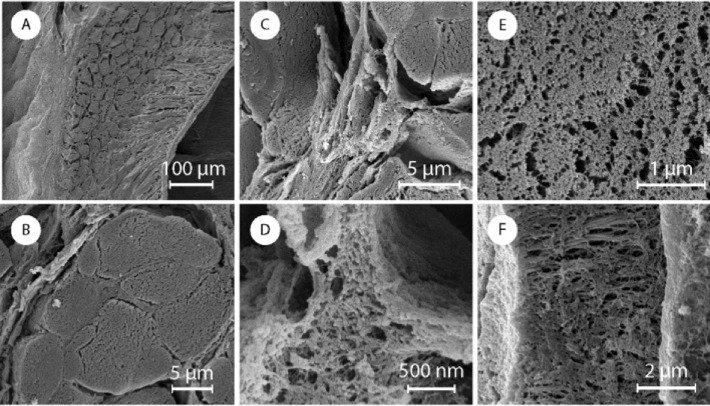
SEM image of a fixed section of the odontophore: (A) ventral coronal section, (B) a fiber perpendicular to the plane of section, (C and D) connecting matrix between the fibers, (E) frontal view of the fiber section and (F) lateral view of the micrometric fiber.

We then examined the odontophore (Fig 1E). The odontophore FTIR spectra has mainly protein absorption bands (Figure I in S1 File). However, weak signals attributed to chitin, such as the asymmetric in-phase ring stretching mode, were also detected. The odontophore dissolves completely in an aqueous solution of 1 M NaOH at room temperature and we do not see any detectable chitin signal from CW staining (Fig 7e and Figure J in S1 File). We thus assume that any presence of chitin is minimal. Identifying the detailed protein composition of the odontophore will be part of future studies. Both in SEM and optical microscope images, the odontophore looked like a continuous single piece from the outside (Figure K in S1 File). The observation of sections, however, unveiled a dense outer layer with aligned perpendicular structures on the inside (Fig 7, and Figure K in S1 File). On a transverse section, Fig 7A and 7D, we observed these structures to be straight lines in the front, and bend into arcs laterally, Fig 7E and 7F. In Fig 7G and 7J we show sagittal sections of the odontophore. In this plane of section we observed straight lines on the ventral region, Fig 7I and 7L, gradually turning into triangles, Fig 7H and 7K, at the dorsal end of the odontophore. With SEM those perpendicular structures appeared composed of ~10 μm thick fibers embedded and connected by layers (Fig 8). Both the fibers and the layers were composed of aligned nanofibrils. A coronal section showed those fibrils perpendicular to the section plane and still aligned, Fig 7M–7R.

We observed these perpendicular structures, composed of fibers, along all the transverse section of the odontophore. On the sagittal section, on the other hand, those structures were present only in part of it, being completely replaced by the dense outer layer in the ventral region of the odontophore, as can be seen in Fig 7G and 7J. The dense outer layer appeared thicker and composed of vertical, poorly fluorescent, fibrous structures. We observed thick, horizontal, highly fluorescent fibers only in the external wall (Fig 7L). The external wall was thicker at the base, getting thinner moving up on the structure and from the anterior to the posterior regions. Its horizontal fibrous composition also seemed to decrease moving from the bottom to the top of the structure leaving space for the vertical fibrous structure. The internal wall, in contrast, maintained a constant thickness along the structure. Higher resolution SEM images confirm the observations from optical microscopy (Fig 8).

## Discussion

We have characterized the structure and composition of several of the acellular components in the buccal mass of *A*. *californicus*, a banana slug. The buccal mass contains the jaw, the radula, and the odontophore as acellular structures. In the buccal mass the radula, supported by the odontophore, works against the anterior part of the jaw in order to cut pieces of food.

We observed that the jaw is directly linked to the foregut cuticle. These two are both made of chitin, but with different morphologies. In the foregut cuticle, perpendicular nanofibrils coil randomly and generate an intricate nanofibrous carpet. In this carpet we observed a sequence of parallel grooves posterior to the jaw. Those grooves have a spacing (41 ± 5 μm), comparable to that of the central denticles [lateral tip-tip distance (45 ± 4 μm)]. We theorize that the radula scrapes against the upper wall of the buccal mass. The protective purpose of the foregut cuticle is known in biology, but its structure has never been characterized. The chitin nanofiber network in the foregut cuticle protects the tissues from damage done by the denticles in the radula. Considering that a denticle’s tip has a curvature of some microns, a randomly coiled nano-fibrous carpet is perceived by the denticle tip as a homogenous surface. Below this structure, the very organized perpendicular fibrils may act as mechanical shock absorbers to the impact of the radula on the tissues underneath.

The dense nanoparticle structure of the jaw, connected by fibrous crosslinks, resembles composite structures and may be responsible for the stiffness of the material. Moreover, the micro-fibrous packing observed from CW staining might contribute to connect the nanoparticles on a bigger scale, mechanically reinforcing the structure. Finally, the darker anterior layer morphology guarantees a homogenous surface for interaction with the denticle’s tips, acting, probably, as a protective layer for the nanoparticles underneath. It is still premature to discuss the origin of the jaw coloration, but we may infer that the color gradient is strictly related to the nanoparticle presence, except in the very anterior layer, meaning that, it is directly associated with the interaction or composition of the nanoparticles.

The function of the radula is to bite off bits of food by working against the jaw. The observation of over 20 different radula samples showed that not one was ever missing one single denticle. Starting from this simple observation we assumed the denticles have a strong anchoring to the membrane and a good ability to disperse mechanical stress that avoid denticles to be pulled out during the biting. The central denticles overlap part of the posterior base of the anterior denticle. Thanks to this interlocking, during biting each denticle compresses the anterior one against the membrane, diminishing the possibility that it will be dragged away. The lateral denticles, instead, have a hook structure that fits on the posterior wall of the anterior denticle. The radula assumes an arc conformation on the odontophore. Senseman[49] described movements of the radula during biting, in *A*. *californicus*, as being largely the result of the action of muscles attached to the odontophore. These muscles produce a cycle of events during a bite[49], which begins with the medial tooth being driven into the food substrate. This is followed by protraction of the radula out of the buccal cavity and onto the food, and then retraction of the radula into the buccal cavity, still moving in an anterior-dorsal arc. A bit of food is scooped out of the substrate during the retraction phase. The lateral denticles act as a serrated cutting tool as the radula moves into and through the surface of the food. Towards the end of the retraction process the lateral denticles scrape against the medial tooth. Senseman suggested that this last phase of biting is functionally equivalent to occlusion in vertebrate biting and serves to sever the bit of food cleanly from the substrate[49]. Therefore, the lateral denticles may also experience a lateral force during the cutting movement. A deeper interaction with the membrane, due to a higher surface of contact, and a partial lateral interlocking between the hook and the gap might be adaptations to face this lateral stress. Moreover, their high inclination may let them experience differently the force of food penetration that might be partially dissipated on the successive denticle.

The results suggested the denticles are mainly composed of organic material reinforced with an additional internal calcium phase, which was not detected as a mineral one by surface vibrational spectroscopies.

The network of radular membrane nanofibrils was mainly aligned longitudinally with the radula, probably to better resist to elongation. The alkaline resistance of those fibers supported the idea that they were chitin-based, with a protein interconnection among fibrils. The denticles have a high fiber content. Probably nanofibrils connect denticle and membrane generating linkages between those two structures. The layered structure observed in cross-sections of the membrane could provide the lower layers the possibility to deform more than the ones interfacing the denticles. This would help interaction of the hard denticles with the soft tissues underneath.

The literature[24] on radula biogenesis and migration reports a tissue between the radular membrane and the odontophore. The role of this tissue is to anchor the radula and move it in order to expose new denticles during the lifespan of the animal. In this study we did not focus on cellular structures or tissues. Despite that, the existence of this tissue explains why we did not detect anchoring components between the radula membrane and the odontophore.

The odontophore works as support for the radula and is strongly linked to the surrounding tissues. As consequence of the strong relationship between odontophore and radula those structures are subjected to similar mechanical stresses.

**Fig 9:**
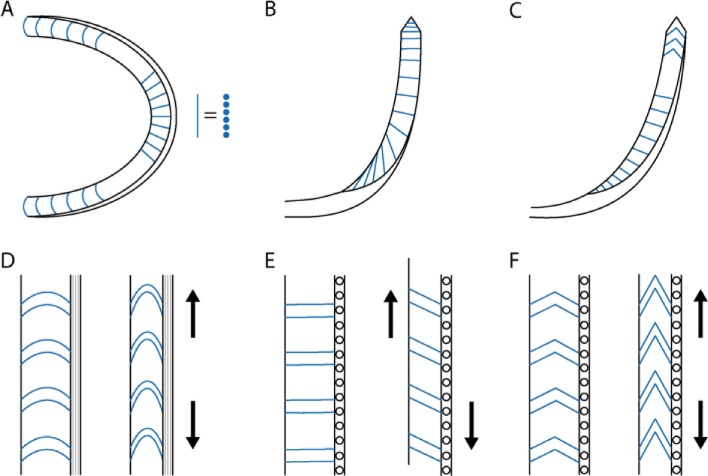
Schematic representation of the odontophore sections: (A) transverse section, (B) coronal section, and (C) sagittal section. Schematic representation of the elastic behavior on different position of the odontophore, the forces inducing the deformation are reported as arrows: (D) transversal posterior region, (E) ventral sagittal region, and (F) dorsal sagittal region.

Microscope images showed that this structure appeared as a continuous nano-fibrous surface from the outside (Figure L in S1 File). Observation of sections, on the other hand, showed a perpendicular fibrous structure with different curvatures inside, schematically represented in Fig 9. This morphology fits with a weakly flexible structure on the ventral sagittal region, which, thanks to its structure, became horizontally elastic (along the transverse plane of section) on the posterior transverse regions, and vertically elastic (along the sagittal plane of section) in the dorsal sagittal region. The perpendicular structures observed in the sections were made up of a single row of microfibers, aligned and connected with the others. A dense layer was observed to cover the whole structure and occupy the area of the odontophore, where the biogenesis and degradation of the radula takes place.

The odontophore’s structure suggests that it would allow an elastic reaction to feeding stresses without excessive surface deformation, avoiding possible tissue damage. The vertical dimension of the jaw was about 800 μm. Comparing this with the odontophore, it shows that only the portion of the radula on the flexible dorsal sagittal region of the odontophore can get in contact with the jaw. At most, 15 denticle rows should be involved in this contact. From the observational evidence collected we could theorize the odontophore could be structurally programmed to drive a correct motion of the radula on the jaw and, meanwhile, dissipate gently the mechanical energy on the surrounding tissues.

## Conclusions

In this work we performed an overall characterization of the three main acellular components in *A*. *californicus*’s buccal mass. For the first time, we characterized the radula of an herbivorous terrestrial slug, and micro- and nano-structures in the jaw and odontophore. The results obtained showed a strong interaction among those structures In the feeding motion both the jaw and radula have a first contact between the compression-resistant nanostructured anterior layer of the jaw and the radula region located on the internal wall of the flexible dorsal region of the odontophore. Although not many rows of denticles are involved in the interaction, their strict interlocking allow them to efficiently disperse the force applied. We observed how both the denticles’ packing and morphology, and the odontophore structure vary between the central and lateral regions, which are subjected to different forces. The odontophore’s structure suggests it produces the correct motion of the radula on the jaw. It also seems to allow proper dispersal of the forces on the surrounding tissues without inducing external deformation that would damage the tissues between the radula and the odontophore. After the first contact between radula and jaw the radula gets in contact with the foregut cuticle, which appeared to be organized to protect the upper foregut.

## Supporting information

S1 File FTIR spectra of α-chitin in blue, β-chitin in red, the peritrophic membrane region in violet, and the jaw in green. The assignment of the absorption band is reported in Table C. As can be observed the β and α polymorph of chitin differ at the amide signal at ≈1600 The spectra present a single peak in β-chitin and two peaks in α-chitin for the amide signal; in γ chitin the two peaks appear of different intensity while usually in α-chitin they have the same intensity **(Figure A)**. Fluorescent images of jaw sections: on the left an unstained control sample, on the right a CW stained sample **(Figure B)**. SEM a fixed jaw section. (A) The transition between the darker frontal layer and the nanoparticles in the jaw. (B) A higher magnification on the frontal layer **(Figure C)**. (A) Dark field back-scattering spectra and (B) dark field forward-scattering spectra of the jaw **(Figure D)**. SEM image of central to lateral denticles conversion in a radula **(Figure E)**. FTIR spectra of banana slug radula teeth in comparison with α-chitin and β-chitin. The assignment of the absorption band is reported in Table C. The β- and α- polymorph of chitin differ at the amide I wavenumber at ≈1600 **(Figure F)**. Fluorescent images of a radula sections: on the left an unstained control sample, on the right a CW stained sample **(Figure G)**. SEM image of the radula membrane. Radula treated in NaOH 1M, A and B, and pristine radula, C and D **(Figure H)**. SEM image of the triangular end on the radula and its denticle-free lateral membrane **(Figure I)**. FTIR spectrum of the odontophore **(Figure J)**. Fluorescent images of a horizontal section of the odontophore: on the left an unstained control sample, on the right a CW stained sample **(Figure K)**. SEM image of fixed sections of the odontophore: (A) upper and (B) lower vertical frontal section, (C) frontal and (D) lateral horizontal section and (E) upper and (F) lower vertical lateral section. External surface of the odontophore, (G) inside and (H) outside the odontophore sphere **(Figure L)**. EDS-SEM analysis of the jaw **(Table A)**. EDS-SEM analysis on the radular teeth **(Table B)**. Assignment of the vibration bands of chitin samples **(Table C)**.
(DOCX)
