# Supplementary material for: Structural characterization of the buccal mass of Ariolimax californicus (Gastropoda; Stylommatophora)
Source: PLoS One. 2019 Aug 7;14(8):e0212249. doi: 10.1371/journal.pone.0212249 (PMC6685607; doi:10.1371/journal.pone.0212249)
Supplement: S1 File — The assignment of the absorption band is reported in Table C. As can be observed the β and α polymorph of chitin differ at the amide signal at ≈1600 The spectra present a single peak in β-chitin and two peaks in α-chitin for the amide signal; in γ chitin the two peaks appear of different intensity while usually in α-chitin they have the same intensity (Figure A). Fluorescent images of jaw sections: on the left an unstained control sample, on the right a CW stained sample (Figure B). SEM a fixed jaw section. (A) The transition between the darker frontal layer and the nanoparticles in the jaw. (B) A higher magnification on the frontal layer (Figure C). (A) Dark field back-scattering spectra and (B) dark field forward-scattering spectra of the jaw (Figure D). SEM image of central to lateral denticles conversion in a radula (Figure E). FTIR spectra of banana slug radula teeth in comparison with α-chitin and β-chitin. The assignment of the absorption band is reported in Table C. The β- and α- polymorph of chitin differ at the amide I wavenumber at ≈1600 (Figure F). Fluorescent images of a radula sections: on the left an unstained control sample, on the right a CW stained sample (Figure G). SEM image of the radula membrane. Radula treated in NaOH 1M, A and B, and pristine radula, C and D (Figure H). SEM image of the triangular end on the radula and its denticle-free lateral membrane (Figure I). FTIR spectrum of the odontophore (Figure J). Fluorescent images of a horizontal section of the odontophore: on the left an unstained control sample, on the right a CW stained sample (Figure K). SEM image of fixed sections of the odontophore: (A) upper and (B) lower vertical frontal section, (C) frontal and (D) lateral horizontal section and (E) upper and (F) lower vertical lateral section. External surface of the odontophore, (G) inside and (H) outside the odontophore sphere (Figure L). EDS-SEM analysis of the jaw (Table A). EDS-SEM analysis on the radular teeth (Table B). Assignme [file pone.0212249.s001.docx]

Supporting information


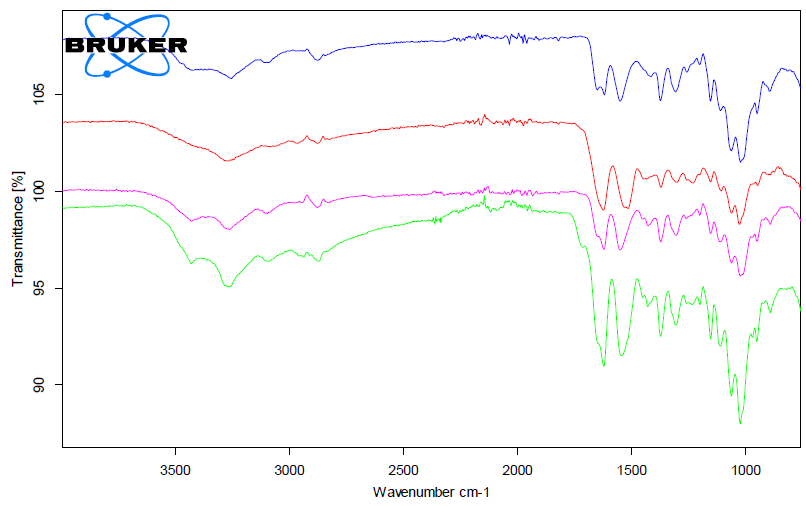


Fig. A


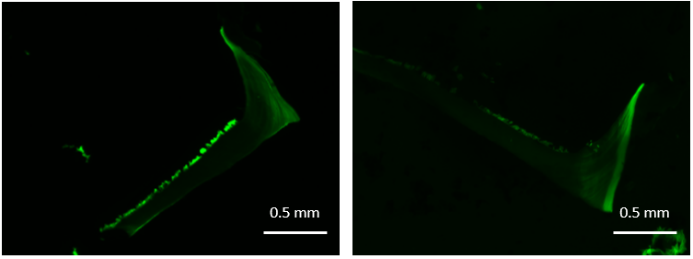


Fig. B


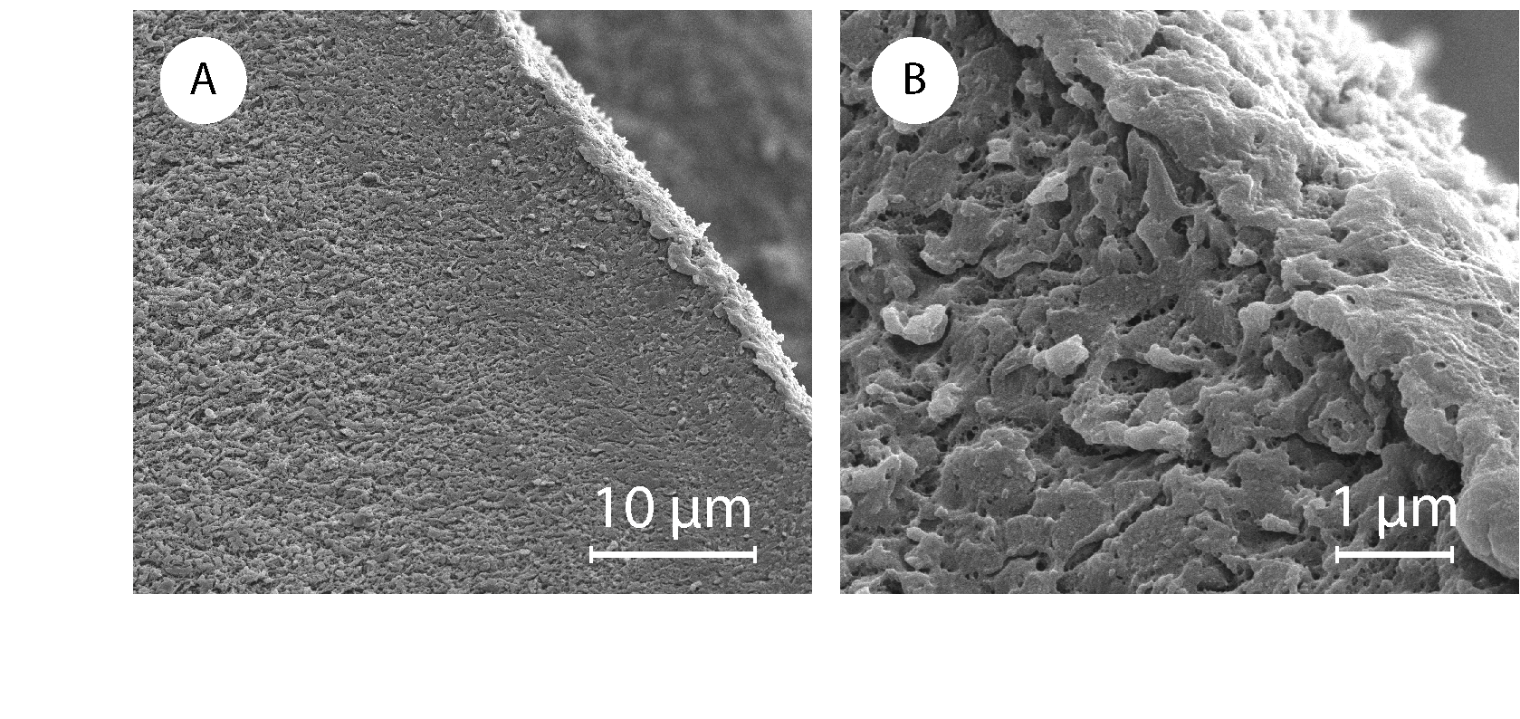


Fig. C


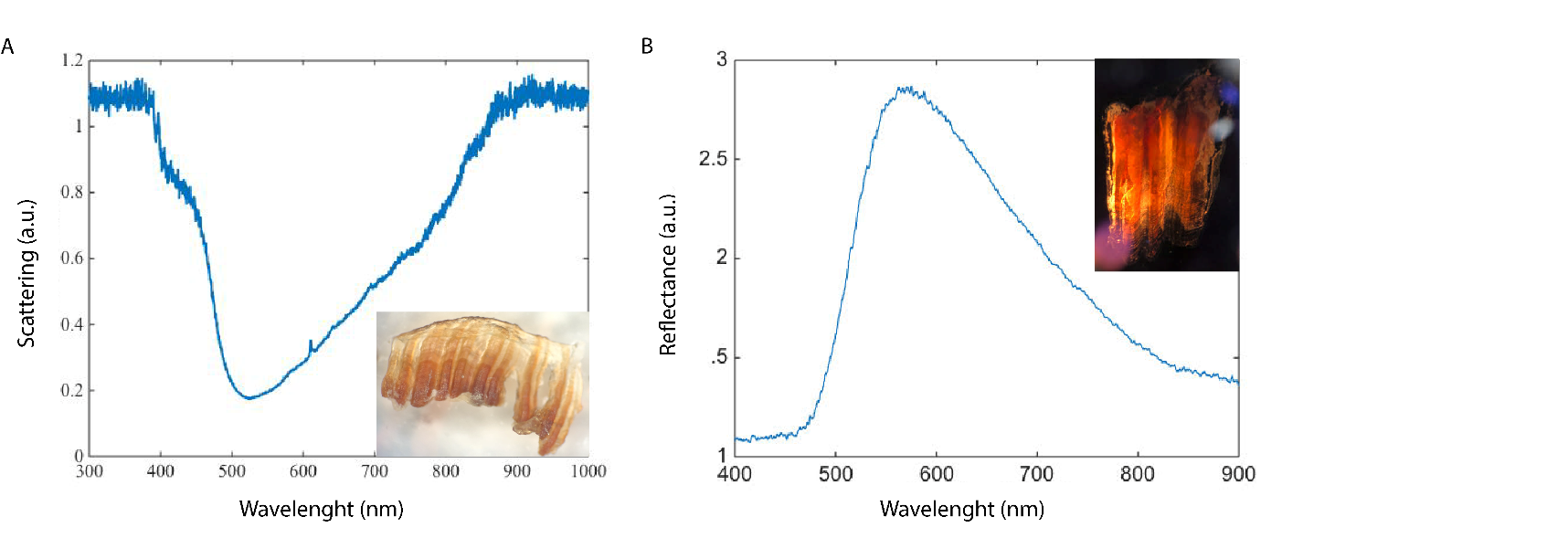


Fig. D


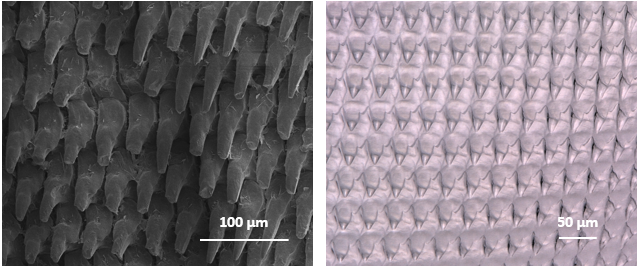


Fig. E


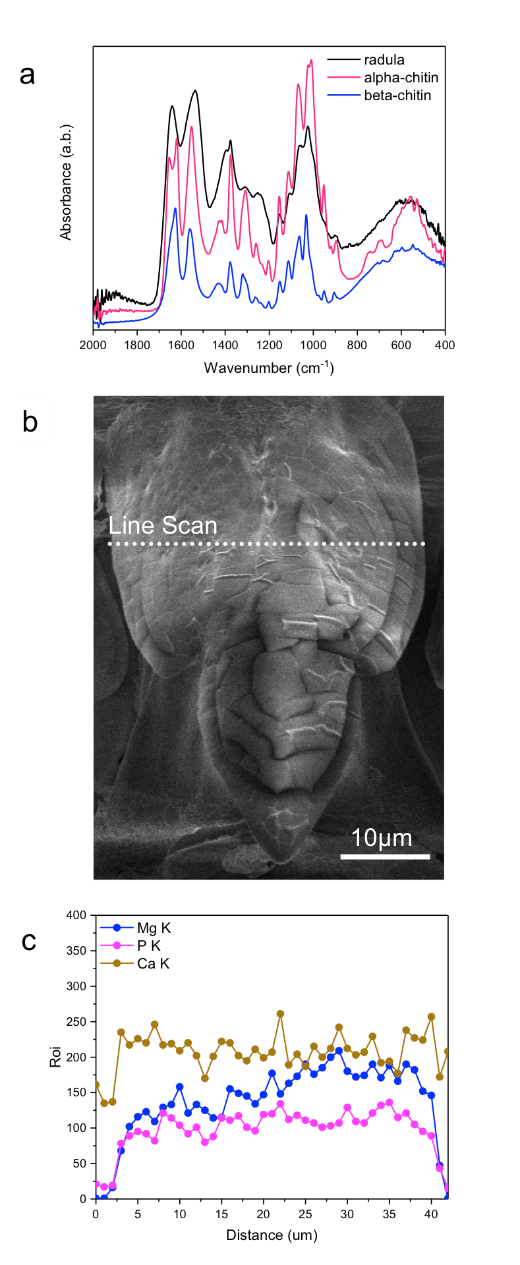


Fig. F


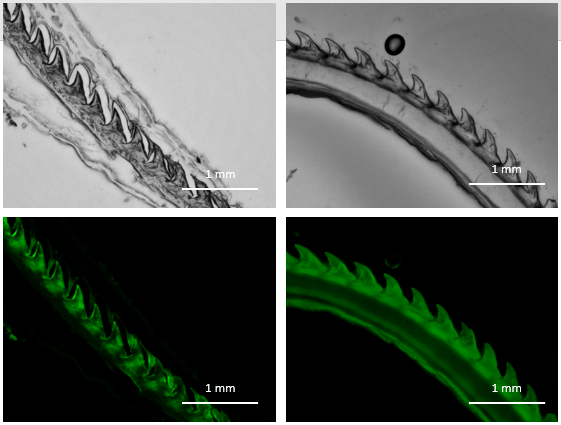


Fig. G.


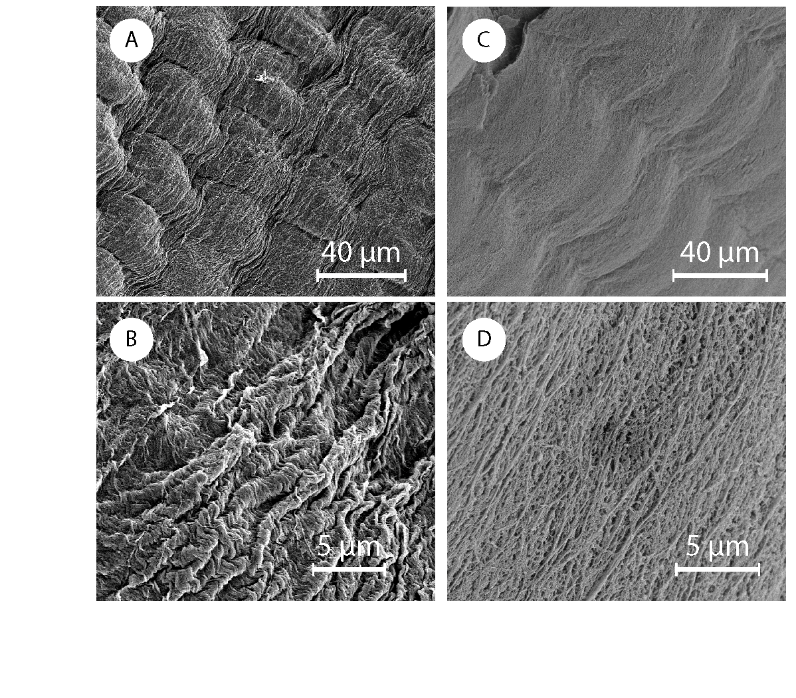


Fig. H


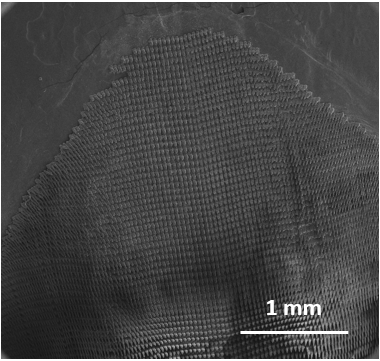


Fig. I


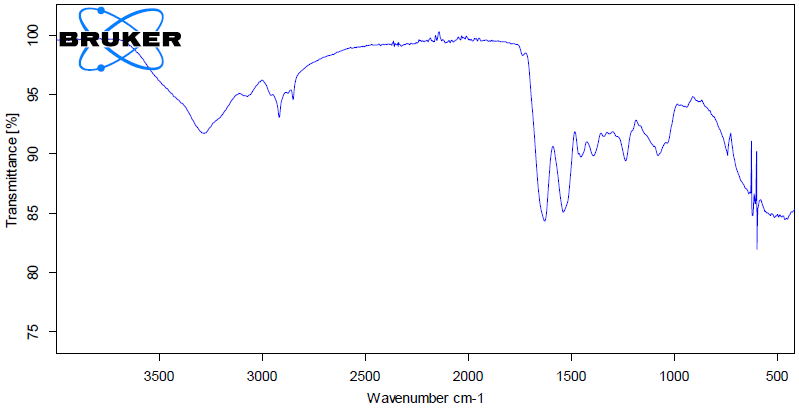


Fig. J


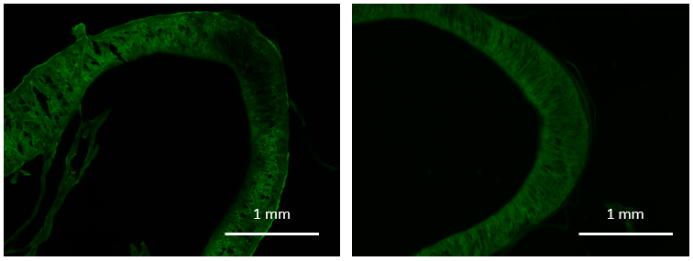


Fig. K


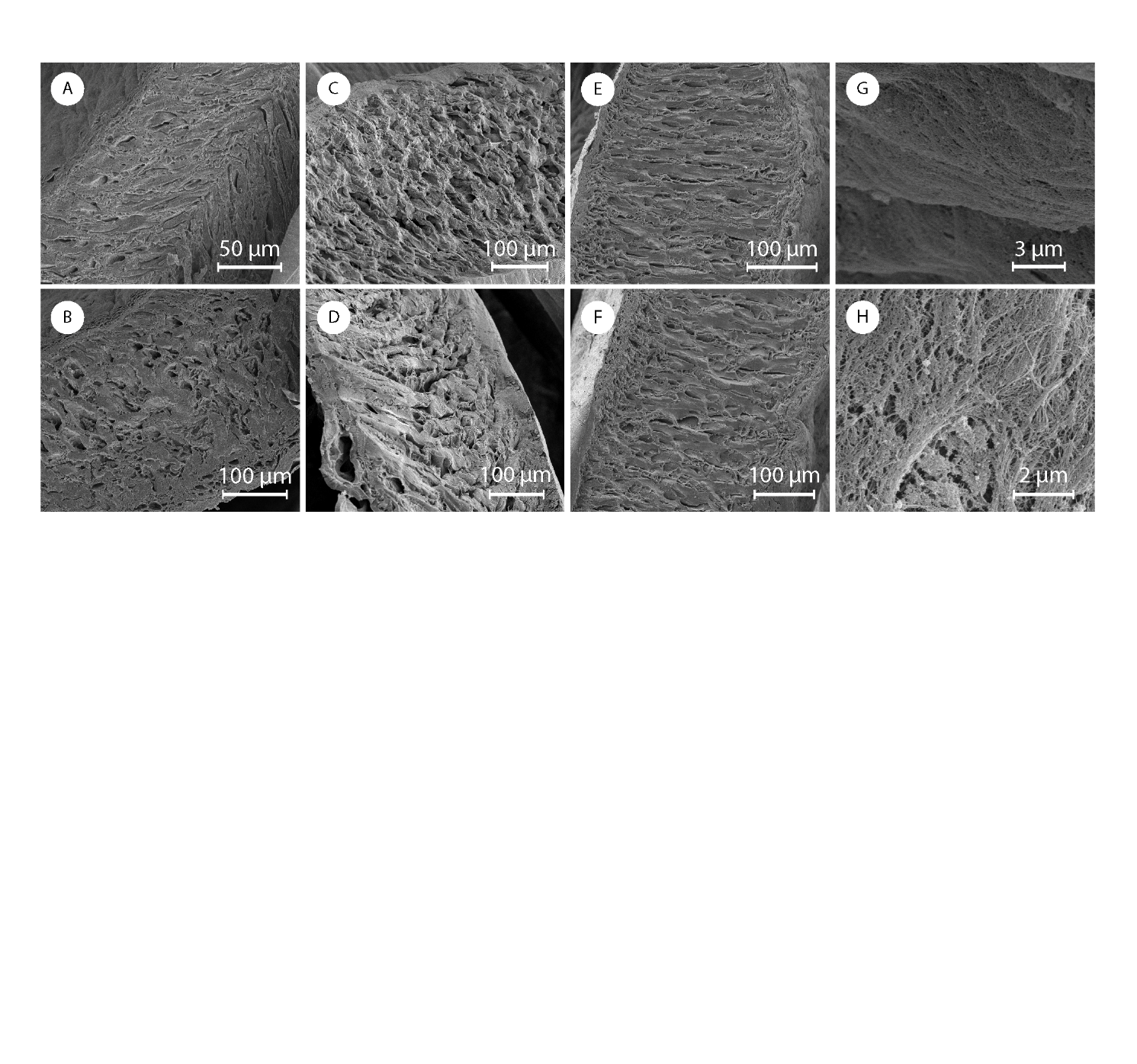


Fig. L

Table A

| **Element** | **Weight %** |
| --- | --- |
| C | 56.2 ± 0.8 |
| N | 9 ± 2 |
| O | 35 ± 1 |
| Na | 0.04 ± 0.03 |
| Mg | 0.1 ± 0.1 |
| Fe | 0.09 ± 0.04 |
| Si | 0.3 ± 0.1 |
| K | 0 |
| Ti | 0.13 ± 0.06 |

Table B

| **Element** | **Weight %** |
| --- | --- |
| O | 66 ± 12 |
| Mg | 5 ± 2 |
| P | 2.8 ± 0.8 |
| S | 4 ± 2 |
| Ca | 9 ± 3 |
| Fe | 0.03 ± 0.03 |

| **Vibration modes^1^** | **A** | **B** | **C** | **D** | **F** | **G** |
| --- | --- | --- | --- | --- | --- | --- |
| OH out-of-plane bending | 690 |  | 687 | 669 |  |  |
| Ring stretching | 899 | 898 | 899 | 898 | 892 | 898 |
| CH_3_ wagging | 944 | 945 | 945 | 945 | 945 | 944 |
| CO stretching | 1034 | 1033 | 1032 | 1036 | 1035 | 1134 |
| CO stretching | 1068 | 1072 | 1066 | 1069 | 1068 | 1169 |
| Asymmetric in-phase ring stretching mode | 1111 | 1115 | 1110 | 1112 | 1111 |  |
| Asymmetric bridge oxygen stretching | 1155 | 1156 | 1155 | 1155 | 1156 | 1154 |
| Amide III | 1202 | 1201 | 1202 |  | 1202 |  |
| band and CH_2_ wagging | 1313 | 1312 | 1315 | 1315 | 1315 | 1315 |
| CH bending and symmetric CH_3_ deformation | 1377 | 1375 | 1377 | 1377 | 1377 | 1377 |
| CH_2_ bending and CH_3_ deformation | 1421 | 1435 | 1421 | 1420 | 1427 | 1424 |
| Amide II band | 1555 | 1558 | 1555 | 1555 | 1556 | 1556 |
| Amide I band | 1654 | 1653 | 1654 | 1654 | 1648 | 1648 |
| CH stretching |  | 2851 | 2857 | 2855 | 2851 | 2852 |
| Symmetric CH_3_ stretching and asymmetric CH_2_ stretching | 2884 | 2872 | 2878 |  | 2878 | 2877 |
| CH_3_ stretching | 2931 | 2918 | 2920 | 2920 | 2920 | 2920 |
| NH stretching | 3097 | 3099 | 3095 | 3094 | 3093 |  |
| NH stretching | 3299 | 3304 | 3289 | 3287 |  |  |
| OH stretching | 3406 | 3398 | 3421 | 3427 | 3434 |  |

Table C
